# Brain plasticity in response to artistic and non-artistic training aimed at promoting creativity: How can we enhance creativity and capture the process in neuroscience?

**DOI:** 10.3389/fnhum.2026.1632331

**Published:** 2026-02-05

**Authors:** Anna Arkhipova, Pavel Hok, Petr Janata, Petr Hluštík

**Affiliations:** 1 Department of Neurology, Faculty of Medicine and Dentistry, Palacký University Olomouc, Olomouc, Czechia; 2 Behavioural and Social Neuroscience, CEITEC - Central European Institute of Technology, Masaryk University, Brno, Czechia; 3 Department of Psychology, University of California, Davis, CA, United States; 4 Center for Mind and Brain, University of California, Davis, CA, United States; 5 Department of Neurology, University Hospital Olomouc, Olomouc, Czechia

**Keywords:** art, brain plasticity, cognitive learning, creativity, decision making, functional connectivity, neuro-esthetics, task-related fMRI

## Abstract

Creativity has been consensually defined as an ability to produce novel and original ideas/works, a definition shared both by the general public and among scholars. Since creativity is one of the most important and unique cognitive constructs seen in human beings, ways to enhance creativity have fascinated researchers across a broad range of human knowledge domains - from the arts and the humanities to science and technology. The functional process of creativity has been actively discussed not only in psychology, but also in neuroscience, where research is uncovering its neural correlates. A great amount of neuroimaging research has focused on describing anatomical and functional adaptations in the brain following various types of cognitive learning and training, e.g., classes of visual art or music composition, courses of drawing, calligraphy or playing musical instruments. A consistent underlying mechanism of domain-specific creativity has not yet been revealed due to difficulties in defining creativity or due to lack of generalizability across different modalities. On the other hand, recent studies suggest that there is a relationship between domain-general creativity and functional connectivity in particular brain networks. In this review, we discuss whether there is evidence for brain plasticity induced by training in creativity and associated behavioral changes, as well as whether the observed brain changes are consistent with the studies of neurobiological underpinnings of creativity and the changes induced by cognitive training.

## 1 Introduction

Creativity is one of the most important human-specific constructs. It has been consensually defined as an ability to produce novel and original ideas/works, a definition shared both by the general public and among researchers (Runco and Jaeger, 2012). Creativity is employed not only in the arts, sciences, and industry, but also in many details of daily work and life, thus, high creativity has a big impact on society and quality of human life. Although the neural correlates of creativity have increasingly attracted neuroscientific research over the past decade, the mechanism by which individual creativity is enhanced is still unclear. Hence, the aim of this review is to provide a brief overview of neuroimaging studies unveiling brain changes related to training/learning in creativity.

For this purpose, we first briefly describe the creativity process, the ways to measure it, and the existing studies of the neural correlates of creativity.

Second, we review the cross-sectional and longitudinal studies of brain plasticity associated with cognitive training/learning in creativity. Cognitive training refers to “interventions using cognitive tasks or intellectually demanding activities, the goal of which is to enhance general cognitive ability” (Gobet and Sala, 2023) both in cognitive neuroscience and psychology, while “creative training” is usually defined as “instruction to develop an individual’s capability to generate novel and potentially useful solutions to (often complex and ill-defined) problems” in creativity research (Birdi, 2016). For the purpose of this review, we consider, given the paucity of neuroimaging studies, training as an active process of developing creative skills through practice and exercise irrespective of training goals. Hence, we include studies in trained fine artists, musicians, poets, and scientists, as well as in artistic education at/after school such as visuomotor learning, music training/improvisation (all of which correspond to artistic training) and other (non-artistic) cognitive training. We also deal with methods aimed at promoting creativity.

Finally, we discuss how the learning data can be integrated with neural correlates of creativity and future directions of neuroscientific studies in creativity training.

## 2 Creativity and its neural correlates

### 2.1 Creativity, its types and measurement

The assessment of creativity developed by psychologists includes the characteristics of originality, fluency, flexibility, openness to new experiences, and the ability of divergent thinking (DT) (Guilford, 1950; Drevdahl, 1956). DT refers to a thought process generating various and often unconventional ideas/solutions against a single problem, whereas convergent thinking leads to conventional solutions. DT is frequently examined in neuroscientific studies of creativity, and DT tests (Guilford, 1950; Torrance, 1968) have been recognized as appropriate indicators of creative ability (see Kim, 2008; Runco and Acar, 2012).

Creativity is usually categorized into two types, i.e., domain-specific creativity, which corresponds to high quality works and achievements in a certain domain, such as visual art, music, poetry and science, and domain-general creativity, which refers to a more basic layer of creative thoughts (Sternberg, 2009). Psychometric tests correlating with domain-general creativity include Torrance Tests of Creative Thinking (TTCT) (Torrance, 1968); Alternative Uses Task (AUT) (Martindale and Mines, 1975); and Chain free association tasks (Benedek et al., 2012). To measure domain-specific creativity, various questionnaires on specific creative achievement are administered, or the subjects are given creative tasks from certain domains. The process of creative activity can be divided into generation and evaluation phases (Ellamil et al., 2012; Liu et al., 2015).

### 2.2 Neural correlates of creativity

**FIGURE 1:**
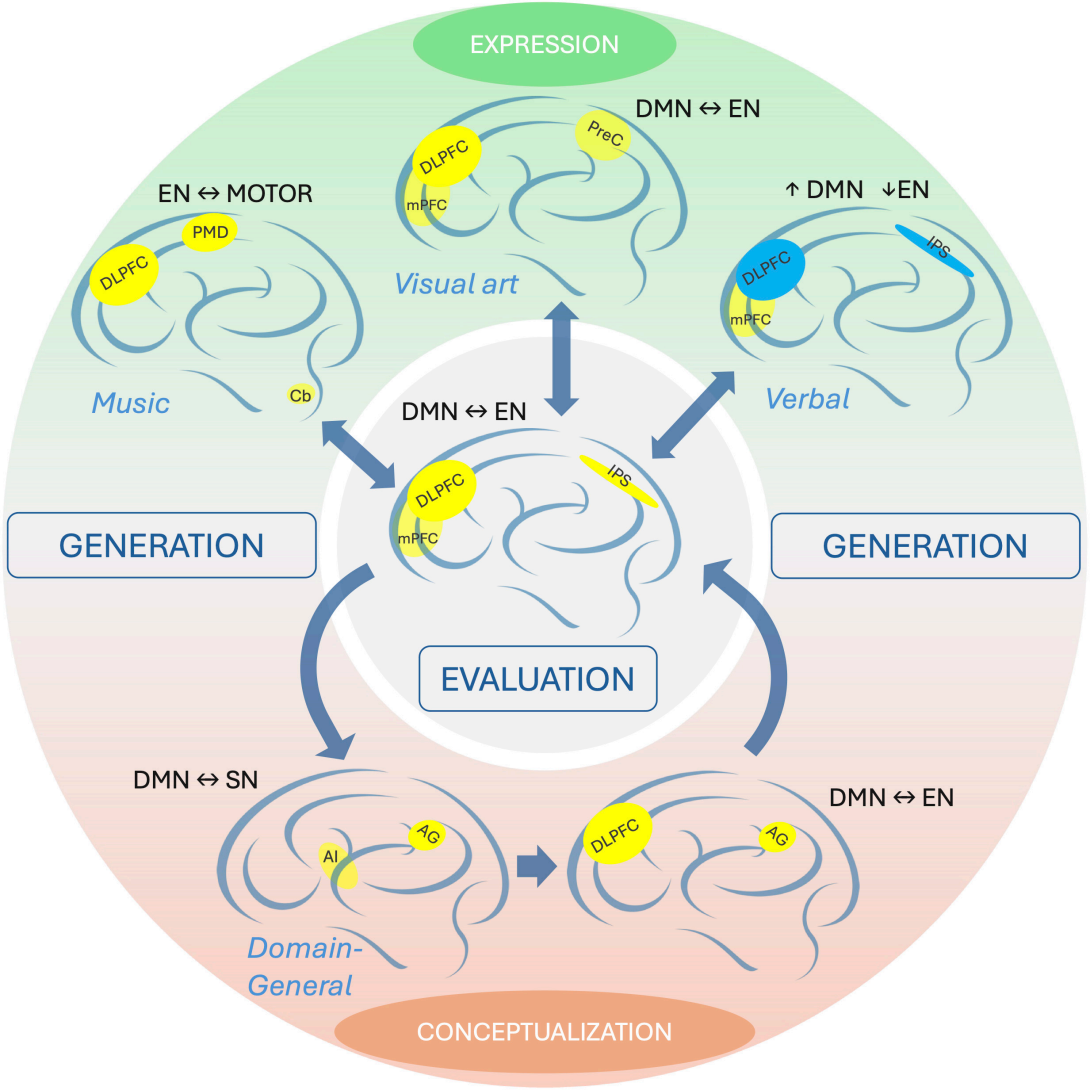
Model of the creative activity process. The creativity process can be divided into generation phase (outer circle) and evaluation phase (center). In a model proposed here, the generation phase can be represented by two activities, conceptualization (bottom) and expression (top). On top of the figure (expression), each brain shows a schematic of the brain network hubs associated with domain-specific creative activity in experts. Yellow regions depict increased activity/stronger connectivity, blue regions depict decreased activity/weaker connectivity according to Pinho et al. (2014) (music improvisation), De Pisapia et al. (2016) (visual art – drawing), Liu et al. (2015) (verbal – poetry). In the center, a brain network pattern during the evaluation phase is shown (Ellamil et al., 2012; Liu et al., 2015). At the bottom, networks involved in domain-general creativity are depicted (Beaty et al., 2017). Bidirectional horizontal arrows in labels indicate functional connectivity between networks. AG, angular gyrus; AI, anterior insula; Cb, cerebellum; DLPFC, dorsal lateral prefrontal cortex; DMN, default mode network; EN, executive network; IPS, intraparietal sulcus; mPFC, medial prefrontal cortex; PMD, dorsal premotor cortex; PreC, precuneus; SN, salience network.

Neuroimaging experiments to investigate neural correlates of creativity have employed various paradigms and tasks, e.g., to study either domain-specific or domain-general creativity and the different phases of the creative process (generation or evaluation). Since a broader overview of neuroimaging methods including EEG has been provided elsewhere (Dietrich and Kanso, 2010; Sawyer, 2011), we focus here on functional MRI (fMRI) as it is the most widely used brain imaging technique. The studies referenced in the text below are summarized in Table 1, representative studies are also visualized in Figure 1 depicting a proposed model of neural correlates of different phases of creative activity process.

**TABLE 1:** List of the reviewed neuroimaging studies, organized by manuscript section.

Section	References	Study design	Participants	Training (intervention/ retrospective)	Creative test/task	Outcome
2.2	Takeuchi et al., 2011	Task-related fMRI	63 young adults (university)	N/A	S-A creativity tests; (in-scanner) n-back task	Creativity correlated with activation in the precuneus (DMN) during the 2-back task
	Benedek et al., 2014	Task-related fMRI	35 young adults	N/A	(In-scanner) AUT vs. recollection	The generation of new ideas involved stronger activation of left IPC; creativity of ideas related to activation of the left IFG (EN).
	Beaty et al., 2015	Task-related fMRI	25 young adults (university)	N/A	(In-scanner) AUT vs. object characteristics task	Hubs of DMN (PCC and right IPL) and EN (DLPFC) showed connectivity during the second half of the DT task
	Marron et al., 2018	Task-related fMRI	36 young adults (university)	N/A	(In-scanner) CFA tasks vs. control cognitive tasks	DMN was more involved in CFA than in other tasks; scores on CFA task were related to higher activation of the DMN and to reduced activation of the left IFG (EN)
	Beaty et al., 2017	Task-related fMRI	35 young adults (university)	N/A	(In-scanner) (creative) metaphor production task vs a synonym production task	Early coupling of DMN (left AG) and SN (right anterior insula) regions and later coupling of DMN (left AG) and EN (left DLPFC)
	Beaty et al., 2018	Task-related fMRI (primary analysis ( validation), rs-fMRI (validation)	163 young adults (university and the surrounding community) and specifically over-sampled art, music, and science majors	Art, music, and science (retrospective)	(In-scanner) AUT vs. object characteristics task	Task-related connectivity and rsFC in high-creativity network (DMN, SN, EN) and low-creativity network (thalamus, DMN, cerebellum) positively and negatively correlated with creativity, respectively
	Boccia et al., 2015	Meta-analysis	508 subjects in total	Mixed	Visuo-spatial, musical and verbal tasks	Distributed brain activations associated with different modalities of creative tasks
	Jung et al., 2010	Structural MRI	61 young adults (university)	N/A	3 DT tasks (free condition of DFT, line condition of DFT and UOT)	High domain-general creativity was associated with a smaller left lingual gyrus and left lateral orbitofrontal cortex as well as larger right PCC and right AG (DMN)
	Chen et al., 2014	Structural MRI	366 young adults	N/A	CAQ and TTCT	CAQ score positively correlated with GMV in anterior DMN nodes; figural TTCT scores correlated with GMV of the left ACC and the medial frontal gyrus
		rs-fMRI				Higher CAQ score inversely associated with rsFC between the dACC and mSFG, right middle frontal gyrus, and left orbito-frontal insula
	Bashwiner et al., 2016	Structural MRI	239 subjects working or studying in the STEM fields	Science, technology, engineering, and mathematics ( music (retrospective)	CAQ	High music CAQ score was positively correlated with cortical surface area/volume in DMN (dmPFC, middle temporal gyrus, and temporal pole)
	Takeuchi et al., 2012	rs-fMRI	159 young adults (university)	N/A	S-A creativity tests	Higher score of S-A tests associated with increased rsFC between the mPFC and the PCC (within DMN)
	Beaty et al., 2014	rs-fMRI	12 in high-creative group and 12 in low-creative group allocated by test	N/A	DT tests (AUT and instances task)	Higher creativity associated with greater rsFC between the left IFG and entire DMN
	Li et al., 2016	rs-fMRI	304 young adults (university)	N/A	TTCT	Higher score in figural TTCT correlated with lower rsFC between DMN nodes (precuneus and mPFC)
	Zhu et al., 2017	rs-fMRI	282 young adults (university)	N/A	TTCT	Higher score in figural TTCT correlated with lower within-network connectivity in the precuneus
3.1.	De Pisapia et al., 2016	Cross-sectional (task-related)	12 professional artists and 12 non-artists	Drawing (retrospective)	(In-scanner) planning artwork and visual imagery of the alphabet	Stronger FC between DMN and EN (precuneus and DLPFC) in artists
	Kowatari et al., 2009	Cross-sectional (task-related)	20 design-major students and 20 non-art major students	At least 2 years of artistic training (retrospective)	(In-scanner) design a new pen	Creativity correlated with the right-hemispheric dominance in the PFC in experts; creativity negatively correlated with activation in the bilateral IPL in novices
	Pinho et al., 2014	Cross-sectional (task-related)	39 pianists	Improvisation and classical piano playing (retrospective)	(In-scanner) music improvisation	The total hours of improvisation experience associated with increased task-specific FC of the bilateral DLPFC; negative association with brain activity in EN
	Andreasen, 2012	Cross-sectional (task-related)	4 professional artists and 3 scientists	Writing, film-making and science (retrospective)	(In-scanner) word association task	Both groups activated the same areas, including DMN
	Liu et al., 2015	Cross-sectional (task-related)	14 professional poets and 13 novices	Writing poetry (retrospective)	(In-scanner) generation and revision of new poetry	Greater deactivation of DLPFC and IPS during generation phase in poets
	Chamberlain et al., 2014	Cross-sectional (structural)	21 art students and 23 non-art students	Attending art and design courses (retrospective)	Questionnaires; observational drawing task	Gray matter density in the right precuneus was correlated with the intensity of art training
	Lotze et al., 2014	Cross-sectional (rsFC)	20 students of creative writing and 23 novices	Attending courses in creative writing (retrospective)	Writing creative text; verbal creativity test	Decreased rsFC between IFG of both hemispheres as well as increased rsFC between right caudate and IPL in experts
	Chen et al., 2019	Cross-sectional (rsFC)	32 calligraphy major students and 44 novices	At least 5 years of training in calligraphic handwriting (retrospective)	Calligraphy performance	In experts shorter characteristic path lengths and higher local efficiency in frontal and parietal cortices, limbic system, basal ganglia, and thalamus
3.2.	Schlegel et al., 2015	Interventional (structural MRI)	17 young adults (university) in active, 18 in control	(Active) 3-month course in introductory drawing/painting; (control) 3-month introductory organic chemistry course	TTCT	Active group increased figural TTCT scores; FA of bilateral prefrontal white matter decreased in art students relative to the controls
	Hyde et al., 2009	Interventional (structural MRI)	15 children in active, 16 in control	(Active) weekly 30 min-keyboard training for a mean interval of 15 months	N/A (finger motor sequencing test)	Improvement in motor ability associated with brain deformation changes in the primary motor area, corpus callosum and primary auditory area
	Habibi et al., 2017	Interventional (structural MRI)	13 children in active, 14 in sports control, 14 in non-sports control	(Active) 2-year 6–7 h weekly music training; (sports control) training in soccer/swimming; (non-sports control) not participating any after-school training	N/A	Different rate of cortical thickening between the right and left posterior STG; higher FA in crossing callosal pathways connecting superior frontal, sensory, and motor segments in active group
	Sachs et al., 2017	Interventional (task-related fMRI)	14 children in active, 13 in sports control, 17 in non-sports control	(Active) 2-year 7h weekly music training; (sports control) training in soccer/swimming; (non-sports control) not participating any after-school training	N/A [(in-scanner) ColorWord Stroop task]	Greater activation within the EN in active group
	Park et al., 2015	Interventional (preliminary, structural MRI)	29 children	15-week training in performing-arts (musical arts or creative movement in music/dance/play)	N/A (Wisconsin Card Sorting Test)	Improved executive function correlated with increased cortical thickness in the left postcentral gyrus and SPL
	Arkhipova et al., 2021	Interventional (task-related fMRI)	22 university students in active, 24 in control	(active) 10.5 h over 2 days workshop of music composing	(In-scanner) listening to different classes of sounds followed by like/dislike decision	Increased favorability toward non-musical sounds; increased activation in DMN and decreased in EN in active group
3.3	Cousijn et al., 2014	Interventional (rsFC)	16 adolescents in active, 16 in active-control	(Active) eight-session of AUT training over 2 weeks; (active-control) eight-session training in rule switching	(In-scanner) AUT	Stronger rsFC between the MTG and bilateral postcentral gyrus associated with better DT performance across both groups
	Wei et al., 2014	Interventional (rsFC)	34 young adults from university	Two stages of AUT training	Verbal TTCT, AUT	Increase in the behavioral measurement of originality; in rsFC between the mPFC and MTG
	Fink et al., 2018	Interventional (rsFC)	24 adults in first training group, 29 in second group	18 verbal DT training modules/units over 3 weeks	AUT, verbal idea generation task	Decreased rsFC within DMN, sensorimotor and auditory networks, as well as in the attention network
	Fink et al., 2015	Interventional (task-related FMRI)	24 adults in first training group, 29 in second group	18 verbal DT training modules/units over 3 weeks	(In-scanner) AUT	Activation changes in the left IPL and the left MTG
3.4.	Zulkifli et al., 2025	Cross-sectional (structural MRI)	675 young adults	N/A (parenting style, retrospective)	S-A creativity tests	Parenting style affects offspring’s creativity correlated with GMV in right SMG (node in SN and DMN)

ACC, anterior cingulate cortex; AG, angular gyrus; AUT, Alternative Uses Task; CAQ, Creativity Achievement Questionnaire; CFA, chain free association; DFT, Design Fluency Test; DLPFC, dorsal lateral prefrontal cortex; DMN, default mode network; DT, divergent thinking; EN, executive network; FA, fractional anisotropy; FC, functional connectivity; GMV, gray matter volume; IFG, inferior frontal gyrus; IPL, inferior parietal lobule; IPS, intraparietal sulcus; mPFC, medial prefrontal cortex; MTG, middle temporal gyrus; rs-fMRI, resting state fMRI; PCC, posterior cingulate cortex; SMG, supramarginal gyrus; SN, salience network; SPL, superior parietal lobule; STG, superior temporal gyrus; TTCT, Torrance Tests of Creative Thinking; UOT, Uses of Objects Test.

#### 2.2.1 Brain activation during the creative process

Several task-related fMRI studies of domain-general creativity indicate the recruitment of modality-independent brain networks, as measured using functional connectivity (FC): the default mode (DMN) and executive-control network (EN) (Takeuchi et al., 2011; Benedek et al., 2014; Beaty et al., 2015; Marron et al., 2018), and the salience network (SN) (Beaty et al., 2017, 2018). Main observations include increased activation and/or higher FC within DMN with suppression in EN, as well as time-varying coupling of both networks. DMN (Raichle et al., 2001) is mostly de-activated when subjects perform a specific cognitive activity and is active during thinking about oneself or others, remembering the past, visceroceptive evaluation and meditation (Raichle, 2015). By contrast, EN is involved in working memory, problem solving, and decision making (Menon, 2011). The suggested role of SN is cognitive control and flexibility through switching between other large-scale networks (Menon and Uddin, 2010). During DT task, Beaty et al. (2015) observed hubs of DMN and EN changing their coupling according to the stage of the task performance. A follow-up study revealed additionally an early coupling of DMN and SN regions (Beaty et al., 2017). This suggests that creative reasoning involves interaction between DMN and EN possibly driven by coupling of DMN and SN. Furthermore, high creativity was associated with FC among the hubs of these large-scale brain networks, suggesting that highly creative people are characterized by the ability to simultaneously engage these networks (Beaty et al., 2018).

On the other hand, a meta-analysis of domain-specific creativity fMRI studies by Boccia et al. (2015) indicated that creativity across investigated domains (visuo-spatial, musical and verbal) involves EN activation, with domain-specific recruitment of the inferior frontal gyrus (IFG) and additional activation in DMN, temporal, parietal, and occipital regions in verbal and musical - but not visual - creativity.

#### 2.2.2 Static brain traits: anatomy and resting-state functional connectivity

Modality differences found in fMRI studies notwithstanding, “static brain traits” associated with individual’s creativity have been found in overlapping brain areas. High domain-general creativity was associated with a smaller left lingual gyrus and left lateral orbitofrontal cortex as well as larger right posterior cingulate cortex (PCC) and right angular gyrus (both nodes of DMN; Jung et al., 2010), whereas higher score in domain-specific Creative Achievement Questionnaire was positively correlated with cortical surface area or volume in the DMN nodes (Chen et al., 2014; Bashwiner et al., 2016).

An association between DMN and high creativity trait was also found using resting-state FC (rsFC): stronger rsFC between nodes within DMN (Takeuchi et al., 2012), greater rsFC between the left IFG and DMN regions (Beaty et al., 2014) or inversely associated rsFC between dorsal ACC and DMN node (Chen et al., 2014) were observed in highly domain-general creative individuals. Oppositely, two studies of (domain-specific) visual creativity found negative correlations between highly creative performance and rsFC: higher score in figural-TTCT was correlated with lower rsFC between the precuneus and medial (m)PFC (Li et al., 2016) and with lower within-network connectivity in the precuneus of the posterior DMN (Zhu et al., 2017). In contrast, high score of verbal-TTCT was associated with decreased rsFC in the mPFC (anterior DMN) while both figural and verbal TTCT were associated with increased rsFC between DMN and EN (Zhu et al., 2017).

Taken together, the domain-general high-creative thinking ability seems to be associated with spontaneous cognitive processes supported by DMN and their increased interplay with high executive functions represented by EN, whilst domain-specific creativity involves (additionally) contribution of more distributed brain regions depending on the task and sensory modality.

## 3 Creativity and brain plasticity in response to cognitive training

### 3.1 “Expert’s brain”: cross-sectional studies in artistic and non-artistic creativity

Experts in specific domains have been often considered as optimal subjects to study the effects of practice/training in creative activities on the brain. The following are examples of studies in which the brain activity of experts was compared with that of novices in task-related fMRI:

1. Planning artwork imagined from a given word was associated with stronger FC between the DMN and EN nodes than visual imagery of the alphabet or rest. The difference was greater in professional artists compared to non-artists. Visual artists may tend to simultaneously activate two processes, i.e., finding and evaluating ideas (De Pisapia et al., 2016).

2. Subjects trained in design and novice subjects were compared during task-fMRI, in which they attempted to design a new pen. In the expert group, creativity was correlated with the right-hemispheric dominance in the PFC, whereas in novices, creativity was negatively correlated with activation in the bilateral inferior parietal lobule (IPL) (Kowatari et al., 2009).

3. Total hours of musical improvisation experience were associated with increased task-specific FC of the bilateral DLPFC, dorsal premotor cortices, and pre-supplementary motor areas, whereas negative association was found with brain activity in EN during performance of improvising task compared with rest, suggesting lower demands on executive control during skilled improvisation (Pinho et al., 2014).

4. No significant difference between artists (writers and a filmmaker) and scientists (neuroscientists and molecular biologists) during a word association task was observed. Since both groups activated DMN and the same areas involved in higher-order socioaffective processing, brain cortices of very gifted artists and scientists may respond similarly (Andreasen, 2012).

5. Generation of new poetry was associated with greater deactivation of the DLPFC and intraparietal sulcus (both part of EN) in experts compared to novices, while the mPFC (DMN) was active during both phases in the both groups. Therefore, experts may prefer being in a defocused state, during which cognitive control is suspended, in order to produce outcomes more efficiently (Liu et al., 2015).

In summary, these studies consistently illustrate the pivotal role of DMN and EN. Creative performance in experts elicited greater activation and FC within DMN, whereas regions of the EN were suppressed/deactivated compared to novices. In contrast, during the evaluation phase, FC between these networks increases in both groups. Thus, long-term training in creative performance may potentially alter the connectivity of these regions.

The central role of cross-modal areas (DMN and SN) has also been demonstrated in studies searching for consistent features in brain structure associated with training in visual art. Increased gray matter density in the right precuneus was correlated with the intensity of long-term training in art school (Chamberlain et al., 2014), whereas gray matter volume of the left anterior cingulate cortex (ACC) and the medial frontal gyrus was correlated with figural-TTCT scores in university students (Chen et al., 2014). Regarding rsFC, Lotze et al. (2014) compared rsFC of creative writers with novices and observed decreased rsFC between IFG of both hemispheres as well as increased rsFC between right caudate and intraparietal sulcus in experts. On the other hand, Chen et al. (2019) compared rsFC of experts in Chinese calligraphy with novices using graph theoretical methods. They observed that the experts showed shorter characteristic path lengths and higher local efficiency in frontal and parietal cortices, limbic system, basal ganglia, and thalamus, concurrently the network measures in their regions were associated with their performance of calligraphy both in copying and creating works.

Cross-sectional studies, however, may not fully differentiate between inherent creativity traits and the effects of training. In the following two sections, we therefore focus on longitudinal studies on cognitive training demonstrating training effects on creative performance and the corresponding brain changes.

### 3.2 Longitudinal studies of brain effects of artistic training

As many examples of creativity training are domain-specific, changes due to increased creative capacity have to be considered with respect to the effects of general artistic training.

Schlegel et al. (2015) observed that a three-month course in introductory drawing/painting resulted in increased figural-TTCT scores in students. DTI analysis showed reorganization of bilateral prefrontal white matter, in which fractional anisotropy (FA) decreased in art students relative to the controls who studied introductory chemistry. Furthermore, the fine-scale multivariate patterns of BOLD activity during this task, especially in the anterior cerebellum functionally related to the right upper limb cortical regions, became increasingly separable between art and non-art students, but no differences using univariate analysis were observed.

On the other hand, general music training, such as weekly keyboard training in children, can lead to improvement in motor ability (finger dexterity) associated with brain deformation changes in the primary motor area, corpus callosum and primary auditory area as measured by structural MRI over 15 months (Hyde et al., 2009). Similarly, comparison of children undergoing 2-year music training with children either involved in sports or not participating in any after-school training revealed a different rate of cortical thickening between the right and left posterior superior temporal gyrus, as well as higher FA in crossing callosal pathways connecting superior frontal, sensory, and motor segments (Habibi et al., 2017). Besides music-specific brain regions, music training may affect the function of other cognitive regions, including the EN. In a longitudinal observational study with a cross-sectional fMRI assessment by Sachs et al. (2017), children with 2-year music training showed greater activation within the EN compared to the control group, while there were no differences in performance on behavioral measures of executive function. On the other hand, in a preliminary study by Park et al. (2015), children after 15 weeks of music training showed improved executive function correlated with increased cortical thickness in the left postcentral gyrus and superior parietal lobule.

Moving to musical creativity training, recently Arkhipova et al. (2021) investigated changes in brain activation during listening to music and non-music sounds after 2-day creativity workshop in which participants composed music using non-music sounds, such as industrial noise and nature sounds. Activation in the trained group increased in DMN and decreased in EN, motor and auditory networks regardless of the sound category, while subjects’ favorability ratings toward non-musical sound samples increased, suggesting that the training broadened aesthetic perception of non-musical sounds. The tendency of change in DMN and EN is similar to aforementioned studies in high domain-general creativity (Beaty et al., 2015; Liu et al., 2015; Marron et al., 2018).

The longitudinal studies in artistic education (Park et al., 2015; Schlegel et al., 2015) therefore suggest that the emergence of creativity and/or concomitant cognitive and artistic skills may be supported by plasticity in neural functions that mediate (possibly domain-specific) sensorimotor integration, whilst more specifically creativity-oriented training, albeit a short one, may lead to changes in the functional brain networks resembling those engaged during the development of domain-general creativity (Arkhipova et al., 2021).

### 3.3 Longitudinal studies with changes in functional connectivity

Another way to capture dynamic changes of brain networks is to investigate functional connectivity within and between functional networks. Several studies (Cousijn et al., 2014; Wei et al., 2014; Fink et al., 2018) have reported modulation of FC after short-term domain-general creativity training. Wei et al. (2014) demonstrated that creativity training induced an increase in rsFC between the mPFC and middle temporal gyrus (MTG) as well as in the behavioral measurement of originality. The effect was larger in individuals who originally had lower creativity. Also, Cousijn et al. (2014) investigated rsFC in adolescents after eight sessions of AUT training over 2 weeks, compared with an active control group. Although they did not find any significant difference between groups, *post hoc* analyses showed that change in DT performance over time course was predicted by FC between the left supramarginal gyrus and right occipital cortex, while stronger FC between the MTG and bilateral postcentral gyrus was associated with better DT performance across both groups. In another longitudinal fMRI study by Fink et al. (2018), successful 3-week verbal DT training measured by AUT was reflected by decreased rsFC within DMN, sensorimotor and auditory networks, as well as in the attention network. Taken together with the related study of task-fMRI after the same training (Fink et al., 2015), in which activation changes were observed in the left IPL and the left MTG, the authors concluded that these global changes in rsFC may support the activity in task-specific brain regions, thus suggesting that the analysis of rsFC can be useful tool for indexing effects of DT training.

As shown above, it can be said that certain educational programs at/after school and short-term training can stimulate creativity and plastic changes in the associated regions in the brain, analogous to plasticity induced by physical exercise or working memory training.

### 3.4 Other methods and means to enhance creativity

Besides specific creativity training, recently Zulkifli et al. (2025) suggest that parenting style affects offspring’s creativity measured by DT as well as brain development in the right supramarginal gyrus, which is a node in the SN and DMN.

Other approaches to enhance creativity include EEG-neurofeedback (Raymond et al., 2005; Gruzelier et al., 2014a, b; Imperatori et al., 2017) and mindfulness meditation (Zabelina et al., 2011; Colzato et al., 2012; Ding et al., 2014) but there are few studies (simultaneously) evaluating the brain changes associated with such interventions [for an overview of effectiveness of creativity enhancement methods in adults, see meta-analysis by Haase et al. (2025)]. For example, an interventional study by Colzato et al. (2012) suggested that mindfulness meditation training improves DT. Meditation is known as a method to reduce stress and promote wellbeing (Zabelina et al., 2011; Colzato et al., 2012; Ding et al., 2014; Brewer et al., 2011) as well as physical and mental relaxation (Deepak, 2019). An fMRI study by Brewer et al. (2011) comparing trained meditators and novices during in-scanner meditation showed that nodes of the DMN (mPFC and PCC) were relatively deactivated in experts. The authors also reported that experience in meditation was associated with decreases in activity in the brain regions comprising DMN during rest, as well as stronger rsFC between PCC and dorsal ACC, and between PCC and DLPFC. The rsFC associated with mediation thus resembles the aforementioned rsFC pattern related to high-creativity prediction model by Beaty et al. (2018).

Taken together with the studies of neural correlates of artistic performance and high creativity mentioned in the first part of this review, it can be suggested that a relaxed state of mind or suppressed self-referential thinking (as reflected by lower activity of DMN during resting state, Brewer et al., 2011), are important factors for one’s best creative performance and decision making. At the same time, effective creativity training seems to promote such patterns in brain activation.

## 4 Discussion and future directions

Based on the aforementioned neuroscientific studies in creativity and its training, one can see that recent studies do not reveal a consistent underlying mechanism across different domains of domain-specific creativity, which may be expected if we conclude that creativity engages domain-specific brain network and areas. On the other hand, studies of domain-general creativity commonly report a correlation with phase-dependent FC, in particular, between/within DMN and EN: Greater deactivation of DMN during rest, stronger activation of the nodes and FC within DMN as well as suppressed EN during generating phase, and simultaneous engagement of both networks during evaluation phase. In fact, even a study using seemingly domain-specific music training, such as Arkhipova et al. (2021), reported brain network changes similar to domain-general creativity training. This could possibly be related to the context of the training. The observed behavioral and brain changes have been interpreted as “broadening” the perception and definition of music sounds and music “elements,” which is similar to the process of divergent thinking. This is in contrast to learning to improvise, for example, wherein the created material typically utilizes the same “set” of music sounds or melodies, harmonies, etc.

The above-mentioned difficulties in the neuroscientific study of creativity may partly be explained by the underlying concepts and definitions of creativity itself. First, one has to keep in mind that creative potential and sense of aesthetics/domain-specific creative elaboration should be considered separately. Even if an individual has high scores in DT, it does not mean that she/he can produce popular art works or innovative scientific evidence. Second, as Csikszentmihalyi (2009) said, “What counts is whether the novelty he or she produces is accepted for inclusion in the domain.” As seen in several studies in domain-specific creativity, the evaluation of creativity was done by experts who are supposed to know how the outcomes are worth/valuable in the domain. Third, in terms of outcomes, a measure of creativity cannot simply be binary. This is clearly visible especially when we consider art works such as Fountain by Duchamp (1917) or 4’33” by Cage (1952), sometimes even the demolition of existing tradition creates a novel/original movement. Taken together, both the methods to enhance and evaluate creativity may require a multidimensional approach.

In future studies in neuroscience of creativity training, we have to be cognizant of whether the design of the specific training aims to develop domain-general or domain-specific creativity, as well as whether the planned assessment of creativity changes is appropriate for that domain. Also, the possibility of “enhancing” creativity through meditation or alpha-theta biofeedback training provides further neuroscientific avenues to creative mind study.
